# Illinois State Board of Health

**Published:** 1883-11

**Authors:** 


The Illinois State Board of Health, which has to grant certificates to practice medicine within the precincts of that State, has just issued an interesting "Report on the Medical Colleges of the Union and of Canada." By the State regulations, these certificates to practice are to be issued only to persons presenting diplomas from medical colleges of "good standing;" and as complaints had been made as to the practice and status of certain colleges, a committee was appointed to determine the requirements and characteristics which constitute "good standing" in a medical college. This committee instituted inquiries into the curriculum and facilities for teaching, the requirements for graduation, fees, etc., of all the colleges in America and Canada, and received official information on all these points, as well as many expressions of opinion as to needed improvements. The standard by which medical colleges are henceforth to be judged in Illinois is certainly none too high. We may summarize the conditions which must be fulfilled as: 1. A preliminary examination in arts. 2. Instruction in anatomy, physiology, chemistry, materia medica and therapeutics, medicine, pathology, surgery, obstetrics and gynæcology, hygiene and medical jurisprudence. 3. Attendance upon two full courses of lectures in different years, each session to extend over five months or nearly twenty weeks at least. 4. Regular attendance upon lectures and "quizzes." 5. The final examinations on all branches to be conducted by other competent examiners than the professors in each branch. 6. Dissection during two courses. 7. Attendance during two terms of clinical and hospital instruction. 8. Professional studies to extend over a period of three years, including the time spent with a "preceptor." As judged by this standard, eighteen medical colleges are rejected, and their graduates will no longer be able to obtain certificates to practice in Illinois, five are accepted "conditionally," and ninety-nine are accepted unconditionally.

The most striking fact elicited by this report appears to be that America is, at any rate, provided with a sufficient number of institutions where students can be taught and graduated in medicine. At present these amount to 110. No fewer than fifty-six others were set going, and, after a more or less successful course, have now become extinct. In Canada nine schools have been established, and are all still at work. Every medical school in the States must possess a license to teach medical sciences from the government of the State in which it is situated, and if power to grant degrees is required, a further license from the same authority has to be obtained. The freedom with which these licenses have been distributed is one of the chief factors in the origin of the multiplicity of schools; and the mere fact of the decay and extinction of such a large proportion of the schools started is quite as striking a proof of the little discrimination with which the power of granting licenses has been used, as are the unsatisfactory provisions for proper teaching and deficient requirements for graduation at some of the colleges. The short life of some of the schools is also evidence in support of the view that such institutions were started with some purpose other than to supply a real want of facilities for medical education.

A consideration of the points in which the American colleges differ from our English schools does not show that they are in advance of us. In a very large proportion, students are admitted to their medical studies without having to pass any examination in general knowledge, or without giving proof of having received a sound general education. This is rightly considered by the Illinois Board as a serious omission, and care is taken to show that nothing is more important for real success in medical studies and practice than a well trained and well furnished mind. We believe that this omission on the part of so many of the American colleges results simply from the pressure of competition. Many of the better institutions refrain from enforcing an entrance examination on their students lest they should be driven to the inferior schools, and so be set free upon the American public worse trained than at present. It seems that the principle of liberty has been carried too far here, and that the need of a responsible central controlling authority to insist upon such an examination being held in all cases is distinctly proved. The period of study, too, is shorter in America than with us. In Canada this is not the case; the curriculum there extends over four years in all cases, and in one college—the Trinity Medical School, Toronto—over four and a half years. In America the period of study is three years, and when we add that one year of these three years devoted to medical study may be spent with a "preceptor," no further proof is needed to show that the training at the colleges must be so hurried as to lack thoroughness and a truly practical nature. In accordance with this we find to a large extent attendance upon "clinics"—clinical lectures—stands in place of daily attendance upon hospital wards, while in the regulations for graduation there is a conspicuous absence of any law requiring the holding of such posts as those of dresser and clerk. It is, however, only by such work that our students learn many of the minor but very important details of medical and surgical practice. Similarly, instruction in practical physiology and histology, and a complete course of dissection, are not insisted on in the majority of these schools. Judging from the facts before us, there is no dearth of lectures, to which the long-suffering students must be subjected the livelong day; but there seems to be a serious want of thorough practical instruction. We are aware that this is made up to a large extent by studies carried on subsequent to graduation, but these are all voluntary on the part of the student, and undertaken only by the more fortunate minority, and we are looking only at the means provided for the general run of the men entering the profession. The last special feature of these "colleges" is that almost without exception they are graduating as well as teaching institutions, and that the lecturers and the examiners are identical. There is much to be urged in favor of allowing the teachers to have some voice in the granting or withholding of diplomas to their pupils, but while admitting this, we cannot but think that the rule of leaving this responsibiltty *solely* in the hands of the teachers is bad for all concerned, and capable of serious abuse. We believe that none are more ready to admit these deficiencies in the medical education of America than the most experienced teachers and practitioners. But to remedy it is by no means easy. Any individual effort in this direction which would be attended by increase in the expense and difficulty connected with taking out a medical qualification, would only have the effect of lessening the number of students at that particular college. Any hearty union for this end between the various competing colleges is almost beyond hope, while, even were this secured, there would be no protection against the establishment of new institutions in which graduation would be temptingly cheap and easy. Some controlling power is needed to insist on a certain standard of teaching and examination—some body that can enforce the institution of a preliminary examination, an extension of time of study, and the acquisition of knowledge by personal observation on the part of the student, rather than by feats of memory. This is contrary to the prevailing spirit in the great Republic, but the nomination of this committee by the Illinois State Board of Health is evidence that the question is exciting attention, and that the solution of the difficulty will be sought for in applying pressure to the teaching institutions from without. In considering critically the medical as all other institutions in America, it is important to bear in mind the short history that country possesses. On all hands it bears the impress of youth, of the failings as well as of the virtues and powers of juvenescence. It is not wise to expect the result of the growth of ages in a Republic of quite recent development. Considering the enormous and rapid growth of this great country, a fact unprecedented in history, the difficulties presented to those who have had to train a sufficient body of medical practitioners have on the whole been overcome with wonderful success. And while now it is easy to point to flaws in the medical education there provided, we may be sure that these will be speedily remedied. And as a set-off to any depreciatory considerations suggested by the report before us, can always be cited the undoubted fact that in no country is the profession held in such general high honor and esteem and treated with so much confidence as in America; and this is the highest reward ever aimed at by honest practitioners, and the best evidence of their real worth.—*Lancet.*

---

WAUKEGAN, ILL, OCT. 2, 1883.

EDITORS OF THE CHICAGO MEDICAL JOURNAL AND EXAMINER,

In the JOURNAL AND EXAMINER for October, page 369, after giving five causes of increased flow of blood to the brain—"gravitation, obstruction, mental activity, physical activity and vasomotor paralysis," I am made to say: "If the hyperæmia depend upon either of the first two it will be arterial," etc., instead of "If the hyperæmia depend upon either of the first two, it will be venous; if upon either of the second two, it will be arterial," etc., as in the manuscript. Please note the correction in the next number of the JOURNAL.

Respectfully,

J. M. G. CARTER, M.D.

